# Supplementary material for: aaquetzalli is required for epithelial cell polarity and neural tissue formation in Drosophila
Source: PeerJ. 2018 Jun 21;6:e5042. doi: 10.7717/peerj.5042 (PMC6015755; doi:10.7717/peerj.5042)
Supplement: Table S1 — Df(3R)Exel6150 and aqzGFP are homozygous lethal, but fully complement insertions in CG9836, CR43130, CG9837 and CG8359. + indicates complementation and ND indicates not determined. The crosses between Df(3R)Exel6150 and mutant alleles produced adults with and without balancers (for PEPIscUG18758(CG9836) = 70 with and 41 without balancers, for PSUPor-PKG08159ry506(CR43130) = 18 with and 82 without balancers and for MiMICCG9837MI01411(CG9837) = 49 with and 81 without balancers). Also the crosses between aqzGFP and mutant alleles produced adults with and without balancers (for PEPIscUG18758(CG9836) = 105 with and 77 without balancers, for PSUPor-PKG08159ry506(CR43130) = 106 with and 64 without balancers, for MiMICCG9837MI01411(CG9837) = 35 with and 53 without balancers and PEPgy2hng2EY18943(CG8359) = 23 with and 50 without balancers). [file peerj-06-5042-s001.doc]

| **Allele**  **(gene)** | ***P{EP}IscUG18758***  ***(CG9836)*** | ***P{SUPor-P}KG08159ry506***  ***(CR43130)*** | ***Mi{MIC}CG9837MI01411***  ***(CG9837)*** | ***P{EPgy2}hng2EY18943***  **(*CG8359*)** |
| --- | --- | --- | --- | --- |
| ***Df(3R)Exel6150*** | + | + | + | ND |
| ***aqz^GFP^*** | + | + | + | + |
