# Supplementary figures and images for: aaquetzalli is required for epithelial cell polarity and neural tissue formation in Drosophila
Source: PeerJ. 2018 Jun 21;6:e5042. doi: 10.7717/peerj.5042 (PMC6015755; doi:10.7717/peerj.5042)

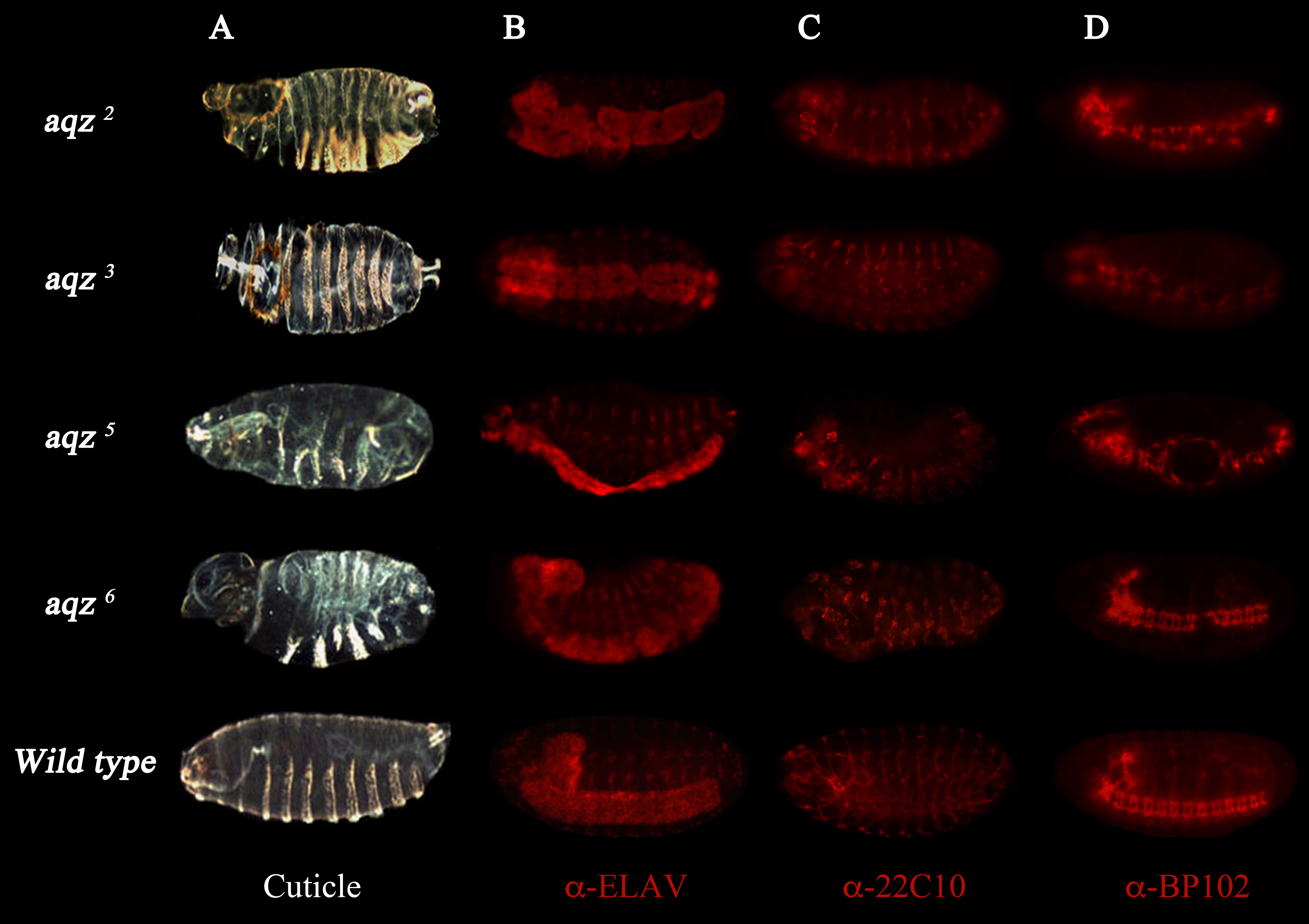

Supplement: Figure S2 — (A) shows cuticular phenotypes; mutant embryos have holes in various positions (dorsal, lateral, anterior), compared to a wild type cuticle (bottom left). (B) shows nervous system staining using the Elav staining, where clear deformities of the central nervous system are shown, compared to the wild type (bottom panel). (C) shows anti-22C10 staining marking neuronal somas and the peripheral nervous system. aqz mutant embryos have irregularities in the disposition of the peripheral nervous system, as well as abnormal regions of low and high density of peripheral nervous system cells, compared to wild type (bottom panel). (D) shows anti-BP102 staining showing abnormalities in aqz mutant connectives in the ventral nervous chord in late embryos (wild type control, bottom panel). All embryos are shown with anterior to the left and dorsal up. [file peerj-06-5042-s003.png]

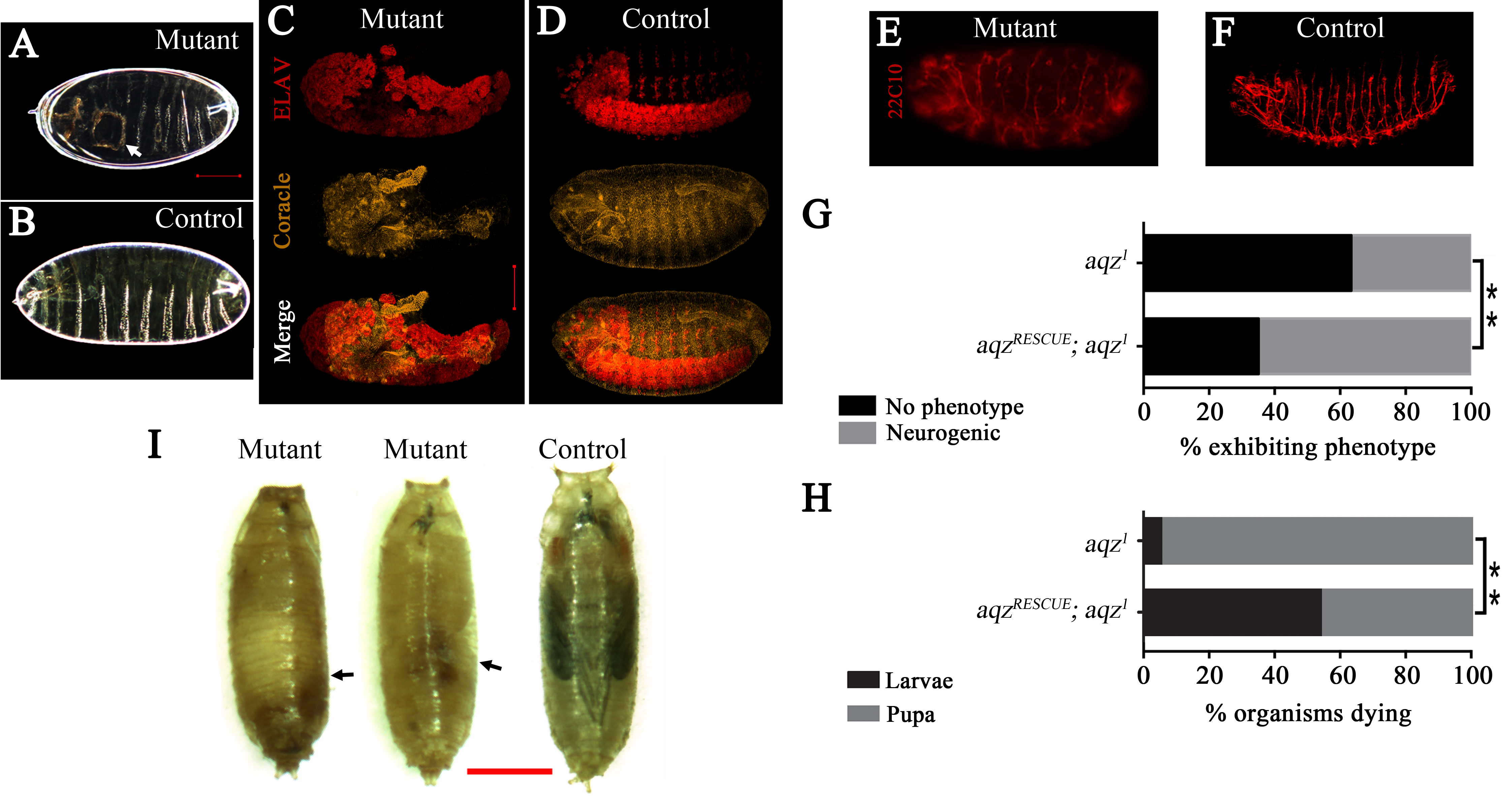

Supplement: Figure S3 — (A) aqz1 mutants have a cuticular phenotype, similar to aqzGFP, but at a lower penetrance. aqz1 cuticles have holes (arrows), compared to (B), a wild type cuticle. Mutant aqz1 embryos have nervous system phenotypes similar to aqzGFP as (C) shows a disorganized and deformed nervous system stained with anti-Elav (top panel), and also shows abnormal epithelial tissue, as marked by anti-Coracle staining (middle panel). Lower panel shows a merged image. (D) shows a similarly stained control embryo. (E) shows an aqz1 mutant embryo displaying abnormal peripheral nervous system, as evidenced by anti-22C10 staining, compared to (F), a wild type control embryo. In all panels, embryos are shown with dorsal up and anterior left. Scale bar are 100 µm. (F) shows a quantification of aqz1 nervous system phenotypes, akin to neurogenic phenotypes, in roughly a third of mutant embryos. Two copies of a genomic rescue construct significantly increase this percentage: over 60% embryos with the rescue constructs exhibit a neurogenic phenotype. (H) The majority of aqz1 embryos that survive embryogenesis die as pupae, with a few (less than 10%) dying as larvae. Adding two aqz genomic rescue constructs significantly increases the number of larvae (over 50%) that do not reach pupation. In (G) and (H) adding wild type copies of aqz increases the aqz1 mutant phenotypes, similar to the effect of two rescue wild type copies of aqz in aqzGFP homozygotes. (I) shows two aqz1 mutant pupae with necrotic patches (black arrows), compared to a wild type control pupa (right). Scale bar 1 mm. Anterior is up. [file peerj-06-5042-s004.png]

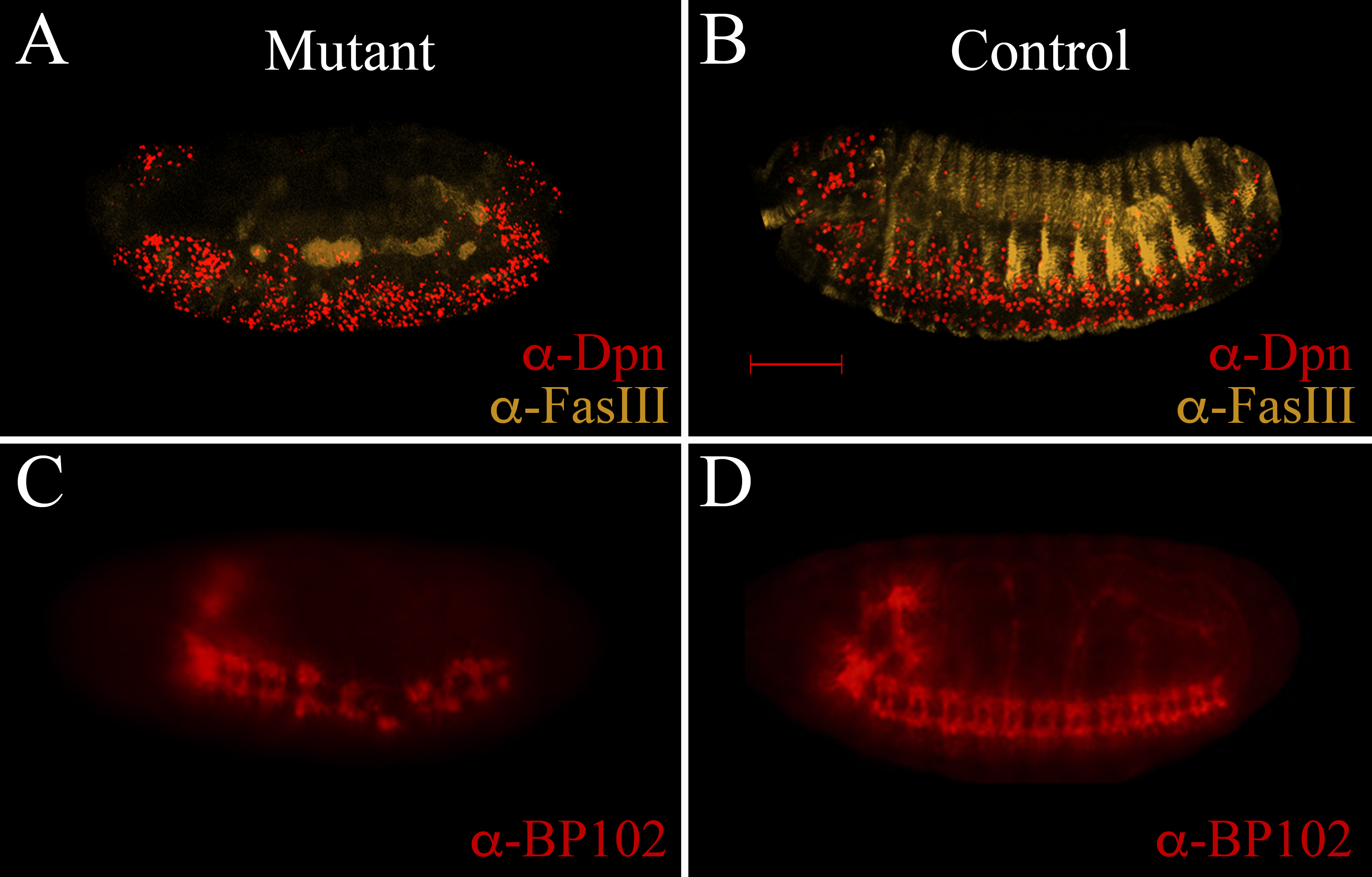

Supplement: Figure S4 — A and C show mutant embryos, while B and D show similarly stained control embryos (heterozygotes). A and B show anti-Deadpan for neuroblasts, and anti-Fascilin III to mark the epithelium. Note disorganization in the mutant embryos. Scale bar is 100 µm. C and D show anti-BP102 staining, showing abnormalities in mutant connectives in the ventral nervous chord. All embryos are oriented dorsal up and anterior left. [file peerj-06-5042-s005.png]

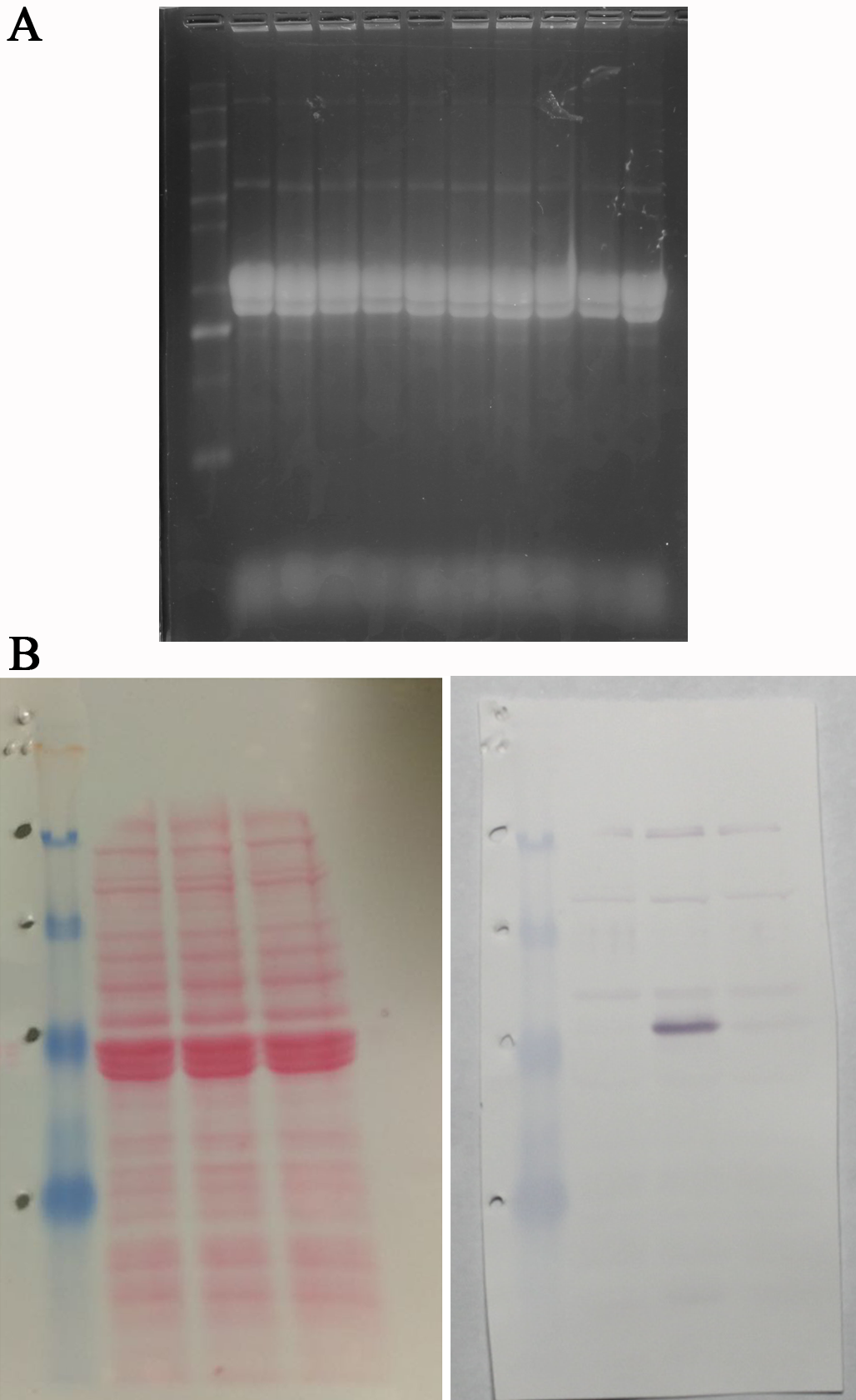

Supplement: Supplemental Information 1 — In (A) is the RNA gel electrophoresis used for the Northern blot shown in Figure 1C. After separating the Drosophila purified RNA, the blot was cut in three pieces (molecular weight markers, and two four lane sections for hybridization) to then probe with the radioactive labelled RNA probes. (B) shows the full Ponceau stained transfer of the electrophoretically separated Drosophila proteins, and in the right the full blot developed with anti-GFP antibodies, and alkaline phosphatase NBT/NCIP substrate. [file peerj-06-5042-s006.png]
