# Supplementary material for: aaquetzalli is required for epithelial cell polarity and neural tissue formation in Drosophila
Source: PeerJ. 2018 Jun 21;6:e5042. doi: 10.7717/peerj.5042 (PMC6015755; doi:10.7717/peerj.5042)
Supplement: Supplementary file 3 [file peerj-06-5042-s002.docx]

SD19655 (3') in pOT2.

```
1	GCAACTCATTGACGGGTTCGGGGTCCACCGGGTGTTCTTGCCATTCATGTCGGTCCTCCAGATCCATCTTCAGATCCAGCTCTAGATCCAGATCCTGTTC
101	CTGCTTTCCTACTCCAGACACTGATCCTCCAGCACCAGTGTGGTTACGACTGCACATTGAGCAGCAACTTTAGGTTGTGCGTGTCGAAGTCATCGAGGAG
201	ATTCCGCTTCGAGCGGCCTGTCTGCATCGGCACCTGGAACTTGGGCATCGACGGCATCATCATGGGAAGCTTACCCTCCGACCTGGACAACGTCCAGTGC
301	TGCGGTGGCTTGGGCAGCGCCGTTGGAGCTGGTGCATCAAAGCACTTGCTGCCCGCGAAATGCGTCAGTGCGGTGGGTATGAGTGGGTGCTGCTGCATCT
401	GGTGCTGGGGCGATATCTTTCCGCCCAACGAAGGGCTCTTCCTCTGCTTGCGAGCCGATCCTCCGACGATTCCCGGCGAGAAACCTCCACCCAAAATGGT
501	CGCTGGTGACTGCCGAACCTGCATAGCTCCCTGGGGCGAACGTCCTCCTCCGTTCCTTCGGTTGTTGTTGGCAAAGAAGAAGCCTCCACCGCCTACAGAG
601	GATCCCTGCTGCTGCTGCGGCGACTGGCGCTGCTGCTGCGACTGCTGATACTTGTTCTTGGATCCGGTCGACTGGGACTGCAGTTGCCGTTGCCTCTGGT
701	TCGGGGGCGTGGCAAGAGCGGATAGACCCAGGCCAAGACCGAGACTCGTGGGCGAGGAGCTCAGGAATTGGGCGTTATTTGTACTGCTGGCAATACTGTT
801	GGAATGTGCAGTTACCAAGGGCGTTGCTGGTGCGGATGCGGATACGGATGTGGGGAGACGGGT    863
```

SD19655 (5') in pOT2.

```
1	AAGCGTACCGATGCGCAAAGAACAAGCATAAGCGAAGAAGATAAAGATATCGATTAAAATTTCTGCTACAAAAATAAATATATATGTAACGCATATTGTA
101	AATGTTCTAAGTTAAGTGACATAAATCAAATATTTGTGTAAAGTTTAATATTTTAATACGCGTCGAAGTACAACGAGTGAGGCTACAGAAGAGCACACAC
201	TAAAGTGGTGTACTTGGCGAGCGCAAATAACGGAAATCAAATTCGAAATAAACGCTGCGCAATAGACGGTGGTGTACATAAGAGTTTAACAAAATCCGAA
301	TCAGAATCAGTTGAAAGTGTGATTTTTTTGAGCCTTTGTCTGATCAGCACGGCTTGGAATGCGAAAGCAGCAGTTAAATCAGCGAAGAACAGAGAGTTTG
401	CGAAATTAAAGCTAAGGATAGCTTAACCGGAGTAAAAAAAAAAATAAATCAACAGACTGATAAACAATACATAACGTTACAAGTAAAACTAAAAGTAAAG
501	TAAACAACAGCTGTACATAAAACATAAAGGAGAAGTGGAAAAGCAGCTACGTGCTCAGCCGCCCGTCGCCCTTTTCGTTTCCATTTTGGAGGTCATCTCA
601	ACTTCGGCGACTATCAGCTACCAAAGAAAATAGAGCGGCGCAGCTAGATCAACATCTCAATAGCAATAGCAGCAGAAGCAGAATCAGAATCAGAAGGCGT
701	TTGGTTTCGATTTATAAAGAATTGATTTAAGTGCGTGTGATATTTAACGTTAAGCATTAATTTAGCGAATTAACCAAAAACAAAAGGGAAAACAGCAGAA
801	ATTTTAATAAGAGAAAG       817
```

RE74095 (3') in pFLC1.

```
1	CAAATTTTAAAAGTTGACTTGGATTTAACACATTTCCAATATTGCTTTACAATTCTTCTCARATTTTTATTCACAATCGTTTCTATAATTACAATATAGA
101	ATTGCGAGTTTGCTCTTCATATTCGCATTCATCTTATGGTCCATTTAAGGTAATTGTAAGGGTATTTAATTAATCGTTTTCTCTTCAGTTGTATTTGTTT
201	TTAATTTGTATACATACCAGGTTTAAAAATATAGCAAAGTATCACAAAAAATTGCAAATTAAATTGTAAATTAGATCAATGGAGAAGTTAAAAAAATACA
301	TAGTGCTTGGTTGCTTTCTTTTCATTGTTTTTAGAACGACGAATCATGTTTCGGCTTAAATAAAAGAGGTTAAAAATAATACTCGAACTTGGTCGAATCG
401	TCGTCGTTACGCTTTAAATTTAAATTAAAAAATTGAATTTGAATTATAATTTCAGAGGCCTTCAATTCGCAGTCGCCTTGGTTGGGGACACAAACGCCTT
501	GGACTCGCAGCATGGAGAGCTCCGCCGCGATCTTGTTTCGGGCTCCCAACATTGAAATGGAAATTATTTTGTACTGATTGGTGTTGAAAAAGTCAGCGGG
601	CCGGAACGTTTGTGCCCACAGTCCAAGGCAGAGCGGGCGAATCTGGGCTGTTTTGGTCTGTTTTAAGTAAAGTGGTTTTCTCTTCTTTTGAATTTTACAA
701	ATAGGTTTTGCATCTGTATCTCTATCCTGCTTCAATGAAATCTAAGCTGTTCTCCGCTTAAACTGACGTCCAGTAAGTAACAAACGTTTTGCTTACACTT
801	AAAAAAAATGCAAAATTCTGCGCGACAAGTTTTGGGTCTTTCTGTGGGG   849
```

RE74095 (5') in pFLC1.

```
1	GGATCGGCTCGCAAGCAGAGGAAGAGCCCTTCGTTGGGCGGAAAGATATCGCCCCAGCACCAGATGCAGCAGCACCCACTCATACCCACCGCACTGACGC
101	ATTTCGCGGGCAGCAAGTGCTTTGATGCACCAGCTCCAACGGCGCTGCCCAAGCCACCGCAGCACTGGACGTTGTCCAGGTCGGAGGGTAAGCTTCCCAT
201	GATGATGCCGTCGATGCCCAAGTTCCAGGTGCCGATGCAGACAGGCCGCTCGAAGCGGAATCTCCTCGATGACTTCGACACGCACAACCTAAAGTTGCTG
301	CTCAATGTGCAGTCGTAACCACACTGGTGCTGGAGGATCAGTGTCTGGAGTAGGAAAGCAGGAACAGGATCTGGATCTAGAGCTGGATCTGAAGATGGAT
401	CTGGAGGACCGACATGAATGGCAAGAACACCCGGTGGACCCCGAACCCGTCAATGAGTTGCAAAAAAAAAATCGATATGAACATAAAACCAACAAAAAAAA
501	ATTAACAAAAACATAAAACAAAAAAATGGAAAATGAAAAAACTTTTACAAAAAAAGCGAAAACATTTCAACTGGAAATTTGAACTTTTTTAAACAAAAAC
601	AAAAAAAAAAAACAAGAGTAATGTTTGTGATCTCAAGTGTGTATCATAAAACAATTAAACCAATTTGAGAAGCTATTAGGTGAGAAGAGAAAAACTGTTT
701	CAAGAAAAGAAAACTGAAAGCAAACAAAATACAAGTAAAAAACACACAAACTACTGTCCTCACACAACCGACCGACAAAAAAGAACATCAACAACGAACG
801	ACAATGAGAA       810
```
